# Requirement for Ergosterol in Berberine Tolerance Underlies Synergism of Fluconazole and Berberine against Fluconazole-Resistant *Candida albicans* Isolates

**DOI:** 10.3389/fcimb.2017.00491

**Published:** 2017-11-29

**Authors:** Yi Xu, Hua Quan, Yan Wang, Hua Zhong, Jun Sun, Jianjiang Xu, Nuan Jia, Yuanying Jiang

**Affiliations:** ^1^Department of Pharmacy, Jinan Military General Hospital, Jinan, China; ^2^New Drug Research and Development Center, School of Pharmacy, Second Military Medical University, Shanghai, China; ^3^Shanghai Pudong Institute for Food and Drug Control, Shanghai, China

**Keywords:** berberine, ergosterol, fluconazole, synergism, tolerance, *Candida albicans*

## Abstract

*Candida albicans* is one of the most common fungal pathogens. Our previous study demonstrated that concomitant use of berberine (BBR) and fluconazole (FLC) showed a synergistic action against FLC-resistant *C. albicans in vitro* and BBR had a major antifungal effect in the synergism, while FLC played a role of increasing the intracellular BBR concentration. Since the antifungal activity of BBR alone is very weak (MIC > 128 μg/mL), it was assumed that FLC-resistant *C. albicans* was naturally tolerant to BBR, and this tolerance could be reversed by FLC. The present study aimed to elucidate the mechanism underlying BBR tolerance in FLC-resistant *C. albicans* and its disruption by FLC. The ergosterol quantitative analysis showed that the BBR monotreatment could increase the content of cellular ergosterol. Real-time RT-PCR revealed a global upregulation of ergosterol synthesis genes in response to BBR exposure. In addition, exogenous ergosterol could decrease intracellular BBR concentration and increase the expression of drug efflux pump genes, further reducing the susceptibility of *C. albicans* to BBR. Similar to FLC, other antifungal agents acting on ergosterol were able to synergize with BBR against FLC-resistant *C. albicans*. However, the antifungal agents not acting on ergosterol were not synergistic with BBR. These results suggested that ergosterol was required for BBR tolerance, and FLC could enhance the susceptibility of FLC-resistant *C. albicans* to BBR by inhibiting ergosterol synthesis.

## Introduction

*Candida albicans* is one of the most common clinical fungal pathogens and causes superficial mycoses, invasive mucosal infections, and disseminated systemic disease (Wilson et al., 2002; Gudlaugsson et al., 2003; Wisplinghoff et al., 2004; Pfaller and Diekema, 2007). Despite the increasing need for effective antifungal therapy, the antifungal agents available are still limited. Fluconazole (FLC), a classic antifungal agent, has been widely used in the clinic due to its high bioavailability and low toxicity (Avmeet et al., 2002; White et al., 2002). However, drug-resistant isolates are emerging rapidly with the increasing clinical use of FLC (Horn et al., 2009). The combination of two or more antifungal agents may be an alternative strategy to solve the aforementioned problem.

Berberine (BBR), an isoquinoline alkaloid, has a long history of medicinal use in traditional Chinese medicine. The biological and pharmacological effects of BBR have been studied extensively in recent years. It was reported that BBR could bind to DNA to affect DNA replication, transcription, and cell cycle (Yadav et al., 2005; Boberek et al., 2010; Bhadra and Kumar, 2011). BBR is used mainly in antibacterial therapy in clinic because of its cytotoxic effect (Stermitz et al., 2000; Ball et al., 2006; Samosorn et al., 2009; Ettefagh et al., 2011). However, the antifungal activity of BBR is extremely weak (MIC > 128 μg/mL) (Nakamoto et al., 1990; Park et al., 1999; Volleková et al., 2003). In our previous study, we found that FLC–BBR combination was highly efficacious in killing FLC-resistant *C. albicans* with the fractional inhibitory concentration (FIC) index was <0.5 (Quan et al., 2006). Our proteomic analysis demonstrated the effect on the expression of proteins functioning in energy metabolism following FLC and BBR treatment. It further revealed that the augmentation of endogenous reactive oxygen species contributed to the synergism of FLC and BBR against FLC-resistant *C. albicans* isolates (Xu et al., 2009). With the deepening of the research, we further found that BBR played a major role in the synergism by causing cell cycle arrest and DNA damage, while FLC played a role of increasing the intracellular BBR concentration by damaging the cell membrane of drug-resistant *C. albicans* isolates (Li et al., 2013). Considering the weak antifungal effect of BBR monotherapy, we hypothesized that a natural BBR-tolerant mechanism might exist in FLC-resistant *C. albicans*, which could be disrupted by FLC.

The present study aimed to elucidate the mechanism underlying BBR tolerance in FLC-resistant *C. albicans* isolates and the effect of FLC on BBR tolerance.

## Materials and methods

### Strains, media, and compounds

Two clinical isolates of *C. albicans* 0304103 and 01010 (both with FLC MIC_80_ > 64 μg/mL and BBR MIC_80_ > 16 μg/mL; MIC_80_ was determined as the lowest concentration of the drug that inhibited cell growth by 80%) were used in this study. *C. albicans* cells were routinely propagated in yeast-peptone-dextrose (YPD) liquid medium (1% w/v yeast extract, 2% w/v peptone, and 2% w/v dextrose) at 30°C in a shaking incubator. FLC (Pfizer-Roerig Pharmaceuticals, New York, NY, USA) was prepared in sterile H_2_O, BBR (Sigma–Aldrich, St Louis, MO, USA) was prepared in dimethyl sulfoxide (DMSO), and ergosterol (Sigma–Aldrich) was prepared in Tween 80–ethanol in 1:1 (v/v) ratio as 5 mM stock (Zhang et al., 2010; Zavrel et al., 2013).

### Quantitative analysis of cellular ergosterol

Total sterols were extracted from whole cells according to a previous report with slight modifications (Liang et al., 2009). Briefly, FLC (64 μg/mL), and/or BBR (16 μg/mL) was added to exponentially growing *C. albicans* cells for 16 h. The same volume of DMSO was added to the control group. The cells were then harvested and washed twice with phosphate-buffered saline [PBS; 10 mM phosphate buffer, 2.7 mM potassium chloride, and 137 mM sodium chloride (pH 7.4)]. The net weight of the cell pellet (approximate 0.5 g) was determined. PBS (2.5 mL) and 6 mL of 15% alcoholic sodium hydroxide solution (15 g NaOH and 10 mL of sterile distilled water, brought to 100 mL with 100% ethanol) were added to each pellet and mixed well by vortexing. The cell suspensions were incubated in an 80°C water bath for 1 h and then cooled to room temperature. Total sterols were extracted thrice with 6 mL of petroleum ether. Sterile distilled water (6 mL) was added to the combined upper phases and swirled for 1 min. Water was removed, and the tubes were incubated in a 60°C water bath until petroleum ether layer volatilized completely. The resulting samples were then dissolved in 0.5 mL of cyclohexane and used for gas chromatography (GC) quantification. Ergosterol standard (0.5 mg/mL), also dissolved in cyclohexane, and the sample-derivatized sterols were run through a VF-5MS fused silica column in a gas chromatograph (Agilent GC 7890B, Palo Alto, CA, USA) interfaced to a flame ionization detector. The injector temperature was 250°C. Moreover, the oven temperature was programmed to constant 100°C for 1 min, followed by a temperature increase of 10°C/min to a final temperature of 300°C. This final temperature was maintained for 10 min. Helium was used as the carrier gas. The linear velocity was 1 mL/min with a split ratio 10:1. The results were obtained by calibrating from the standard curve of ergosterol.

### Real-time RT-PCR

The hot-phenol method was used to extract total RNA samples from *C. albicans* isolates (Schmitt et al., 1990), which were treated with DNase I (TaKaRa Biotechnology, Dalian, China) to remove genomic DNA contamination. Reverse transcription was performed in a total volume of 20 μL with Avian Myeloblastosis Virus Reverse Transcriptase (TaKaRa Biotechnology), Random Primer (6-mer) (TaKaRa Biotechnology), and 1 μg of total RNA, followed by an exposure to 30 °C for 10 min, 45°C for 15 min, and 99°C for 2 min, as recommended by the manufacturer.

Real-time RT-PCR reactions were performed with SYBR Green I (TaKaRa Biotechnology), using a LightCycler Real-Time PCR system (Roche Molecular Biochemical, Mannheim, Germany). Gene-specific primers were designed using the Discovery Studio Gene software (Accelrys, Inc., San Diego, CA, USA). The primers are shown in Table 1. The thermal cycling conditions were as follows: an initial step at 95°C for 10 s, followed by 40 cycles at 95°C for 10 s, 62°C for 20 s, and 72°C for 15 s. The system software was used to monitor the change in fluorescence of SYBR Green I in every cycle, and then the threshold cycle (*C*_T_) was measured. For internal control, 18S rRNA was used. The formula 2^-ΔΔ*C*^T was used to calculated the gene expression level of the drug-treated cells relative to that of the control cells, where Δ*C*_T_ was the *C*_T_-value of genes of interest minus that of the internal control, and ΔΔ*C*_T_ was the mean Δ*C*_T_ value of the drug-treated cells minus that of the control cells.

**Table 1:** Sequences of the primers used in the present study.

**Primer name**	**Sequence (5′-3′)**	**Amplicon size (bp)**
18s	(F) TCTTTCTTGATTTTGTGGGTGG	150
	(R) TC GATAGTCCCTCTAAGAAGTG	
ERG1	(F) TTAGAATCATGCCAAACC	127
	(R) CCAACTGTCATACCACCC	
ERG2	(F) TAATAATGCTGGTGGTGC	167
	(R) CAGGATAAGC TGC TCTTT	
ERG3	(F) GTC TAATGACCCAGTTGT	162
	(R) TCTTCTTCTGCCTTTGCA	
ERG4	(F) TATACGCCAATGCTTGTG	120
	(R) AGTAACTGAATGGAACCC	
ERG5	(F) AGATACCGTCCACCAGTC	119
	(R) TGCAAAGCAGGATACAAT	
ERG6	(F) GCTACCGTTCATGCTCCA	164
	(R) CCATCACCGACTTCAATA	
ERG7	(F) GCTTGGGCTTTGATAGGG	106
	(R) TC CACTCACCAGTCGGTA	
ERG10	(F) TGC C TTGGGTC ATCCT CT	10S
	(R) CCGTTACAAACACCAGCA	
ERG11	(F) GAATCCCTGAAACCAAT	131
	(R) AGCAGCAGTATCCCATC	
ERG13	(F) TGGAACACGCTTAC GATT	191
	(R) CACATGGAAGGCACTGAA	
ERG24	(F) GGTGACTTAGCGTGGGT	143
	(R) GCTGAGCGGAAGATGTA	
ERG25	(F) GCAGCAGAATATGCTCATC C	176
	(R) CCAGAATGAGAATCAACGGC	
MDR1	(F) GCCGATTACAAACCAACTCT	111
	(R) ATCATCATCAC CATC C C AAG	
CDR1	(F) TGAATACCACGGGTTTGATG	116
	(R) TCATGTT CATATGGATTGAC C	
CDR2	(F) AAAAAGGTGGAAGAACGGC	160
	(R) TTGGCATGAGATCCTGGTG	

*F, forward primer; R, reverse primer*.

### Growth curve assay

Exponentially growing *C. albicans* cells were harvested and resuspended in fresh YPD medium to 1 × 10^5^ CFU/mL. Various concentrations of BBR and ergosterol (alone or in combination) were added (Zavrel et al., 2013) to the cells. The cells were cultured at 30°C with constant shaking (200 rpm), and the OD_600_ was measured at designated time points after culture (0, 3, 6, 9, 12, and 24 h). The same volume of solvents (DMSO, Tween 80, and ethanol) was added to the untreated control group. Three independent experiments were performed.

### Intracellular BBR accumulation assay

Intracellular BBR concentration was detected according to a previously described protocol (De et al., 2007; Pereira et al., 2007), with a few modifications. Exponentially growing *C. albicans* cells were harvested, washed twice with PBS, and resuspended in RPMI 1640 medium at a cell density of 1 × 10^7^ CFU/mL. Then, different concentrations of BBR and ergosterol were added. The same volume of solvents (DMSO, Tween 80, and ethanol) was added to the untreated control group. Each sample (1 mL) was cultured at 30°C with constant shaking (200 rpm) for 0, 2, 4, 6, 8, and 10 h; centrifuged; washed twice; and resuspended in 1 mL of PBS at 1 × 10^7^ CFU/mL. Then, 100 μL of each disposed sample was transferred into a black, clear-bottomed, 96-well microplate (Greiner, Pleidelsheim, Germany). An Infinite 200 Universal Microplate Reader (Tecan Group Ltd., Männedorf, Switzerland) was used to measure the fluorescence of BBR at 405-nm excitation and 520-nm emission wavelengths.

### Checkerboard microdilution assay

Assays were carried out in 96-well microtiter plates (Greiner) according to a broth microdilution protocol of the Clinical and Laboratory Standards Institute M27-A3 method, with a few modifications (Quan et al., 2006; Xu et al., 2009; Li et al., 2013). The initial concentration of fungal suspension in RPMI 1,640 medium was 1 × 10^3^ CFU/mL. The final concentrations ranged from 0.125 to 64 μg/mL for FLC and 1 to 32 μg/mL for BBR. The plates were incubated at 35°C for 24 h. MIC_80_s were determined as the lowest concentration of the drugs (alone or in combination) that inhibited growth by 80% compared with that in drug-free wells. The FIC index was defined as the sum of the MIC_80_ of each drug when used in combination divided by the MIC_80_ of the drug used alone. Synergy and antagonism were defined by FIC indices of ≤ 0.5 and >4, respectively. An FIC index of > 0.5 but ≤4 was considered indifferent (Odds, 2003).

### Transmission electron microscopy

Exponentially growing *C. albicans* cells treated with 10 μg/mL of FLC and/or 16 μg/mL of BBR for 16 h were washed twice with PBS and fixed in 500 μL of a fixative solution (sodium cacodylate buffer, pH 7.2, containing 4% polyoxymethylene) at 4°C for 24 h. The samples were then washed with saline and postfixed with 1% phosphotungstic acid for 90 min. The fixed cells were then dehydrated through a graded series of ethanol and embedded in EPON-812. Ultrathin sections were prepared, double stained with 3% uranyl acetate (Biopeony, Beijing, China) and 3% lead citrate (Sbjbio, Nanjing, China), and observed under a transmission electron microscope (Hitachi H-800, Tokyo, Japan) at a magnification of ×10,000.

### Statistical analysis

Data were expressed as the mean ± SD of the independent assays in triplicate. The Student's *t*-test was used to assess the significance of the differences. A *P* < 0.05 or < 0.01 was considered statistically significant.

## Results

### BBR increased cellular ergosterol content in FLC-resistant *C. albicans*

**Figure 1:**
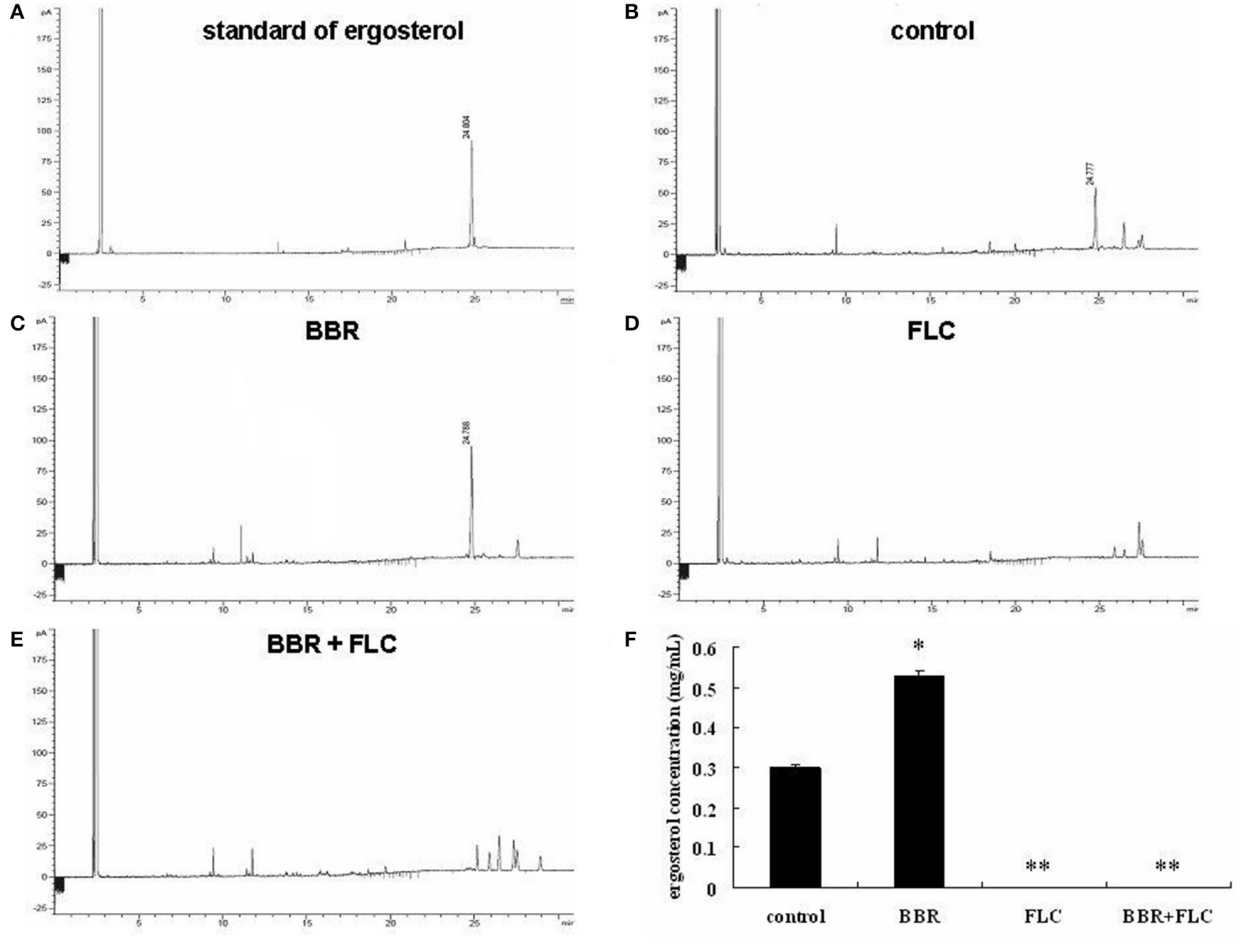
Quantitative analysis of cellular ergosterol by GC in *C. albicans* isolate 0304103 treated with 16 μg/mL of BBR and/or 64 μg/mL of FLC for 16 h. **(A)** Chromatogram of standard of ergosterol (0.5 mg/mL). **(B)** Chromatogram of sterols extracted from untreated cells. **(C)** Chromatogram of sterols extracted from BBR-treated cells. **(D)** Chromatogram of sterols extracted from FLC-treated cells. **(E)** Chromatogram of sterols extracted from BBR + FLC-treated cells. **(F)** Ergosterol content of cells treated with BBR and/or FLC. Values are described as ergosterol (mg/mL). All values are expressed as the mean ± SD of triplicate assays. ^*^P < 0.05 vs. control cells; ^**^P < 0.01 vs. control cells.

In this study, we investigated the mechanism underlying BBR tolerance in FLC-resistant *C. albicans* isolates and the effect of FLC on BBR tolerance. First, the cellular ergosterol level was measured using GC analysis in the FLC-resistant clinical isolate 0304103 after FLC and/or BBR treatment. Interestingly, the ergosterol content significantly increased in the cells treated with 16 μg/mL BBR compared with the control group (0.536 vs. 0.298 mg/mL), but was barely detected in the cells treated with 64 or 64 μg/mL FLC combined with 16 μg/mL BBR (Figures 1A–F). The findings suggested that BBR monotreatment resulted in marked augmentation of cellular ergosterol content in FLC-resistant *C. albicans*. However, this effect could be obliterated by the presence of FLC.

### BBR increased mRNA levels of ergosterol synthesis genes

**Figure 2:**
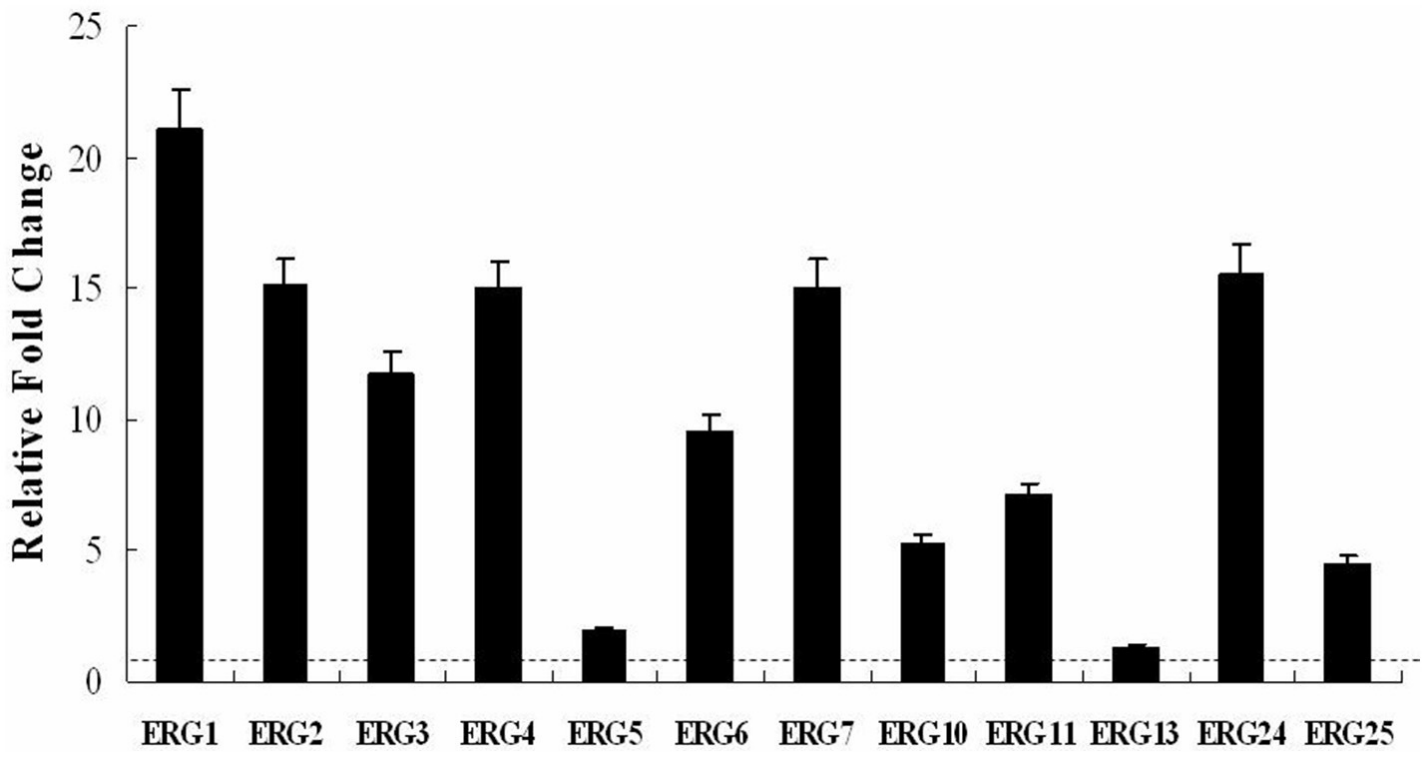
Changes in the expression of ergosterol synthesis genes in *C. albicans* isolate 0304103 treated with 16 μg/mL of BBR for 6 h. All genes were examined by real-time RT-PCR with gene-specific primers. Values represent the mean ± SD of three replicates.

To further investigate the effect of BBR on the expression of ergosterol synthesis genes, the real-time RT-PCR analysis was performed in isolate 0304103 treated or untreated with 16 μg/mL BBR for 6 h. The same volume of DMSO was added in the BBR-untreated group. The results revealed a global upregulation of ergosterol synthesis genes in response to BBR treatment. Figure 2 shows a significant increase (more than 10-fold) in the expression of genes *ERG1, ERG2, ERG3, ERG4, ERG7*, and *ERG24* (11.75- to 21.06-fold), and a modest increases in the expression of genes *ERG5, ERG6, ERG10, ERG11, ERG13*, and *ERG25* (1.35- to 9.50-fold). These results indicated that BBR monotreatment increased the mRNA level of genes related to ergosterol synthesis, which was consistent with the result of ergosterol content measurement.

### Feeding ergosterol reduced the susceptibility of FLC-resistant *C. albicans* isolate to BBR

**Figure 3:**
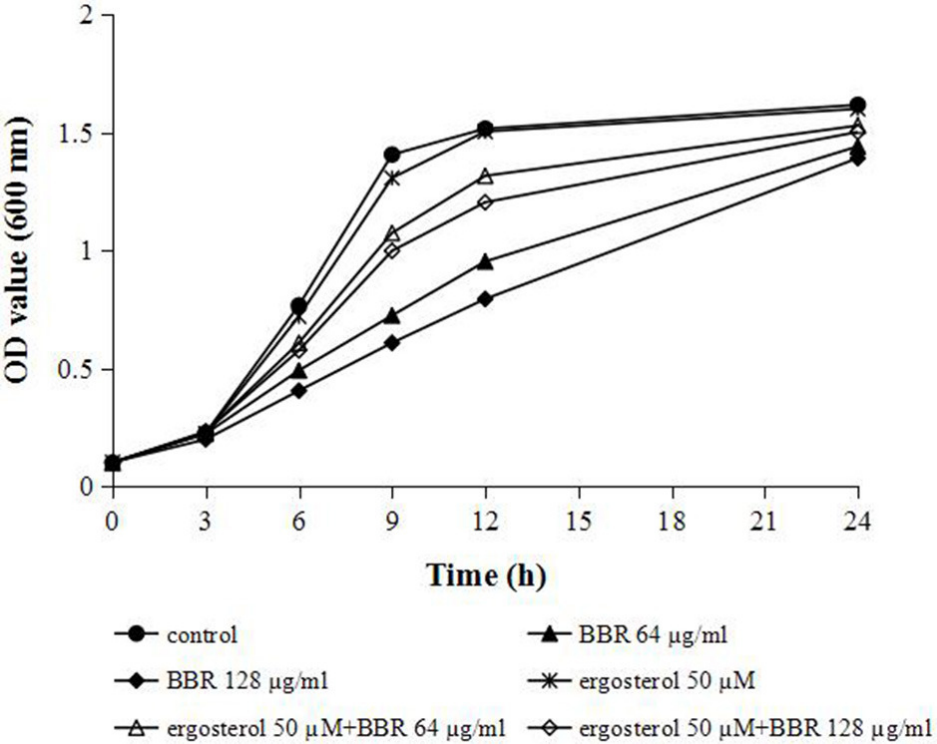
Growth curves of *C. albicans* isolate 0304103 at different concentrations of BBR ± 50 μM ergosterol. A sample was taken to measure the OD_600_ at different times, from which a representative growth curve was obtained.

We hypothesized that the increase in cellular ergosterol content might be involved in BBR tolerance in FLC-resistant *C. albicans*. To test this hypothesis, the effect of feeding exogenous ergosterol on the susceptibility of isolate 0304103 to BBR was examined. The result of growth curve assay indicated no obvious effect on the growth of this isolate treated with 50 μM ergosterol, whereas the growth rates of the cells slowed down significantly when treated with high doses of BBR (64 and 128 μg/mL) (Figure 3). Interestingly, feeding 50 μM ergosterol markedly increased the growth rates of BBR-treated cells (Figure 3), suggesting that the fungistatic effect of BBR can be weakened in the presence of exogenous ergosterol.

### Feeding ergosterol reduced intracellular BBR concentration in FLC-resistant *C. albicans*

**Figure 4:**
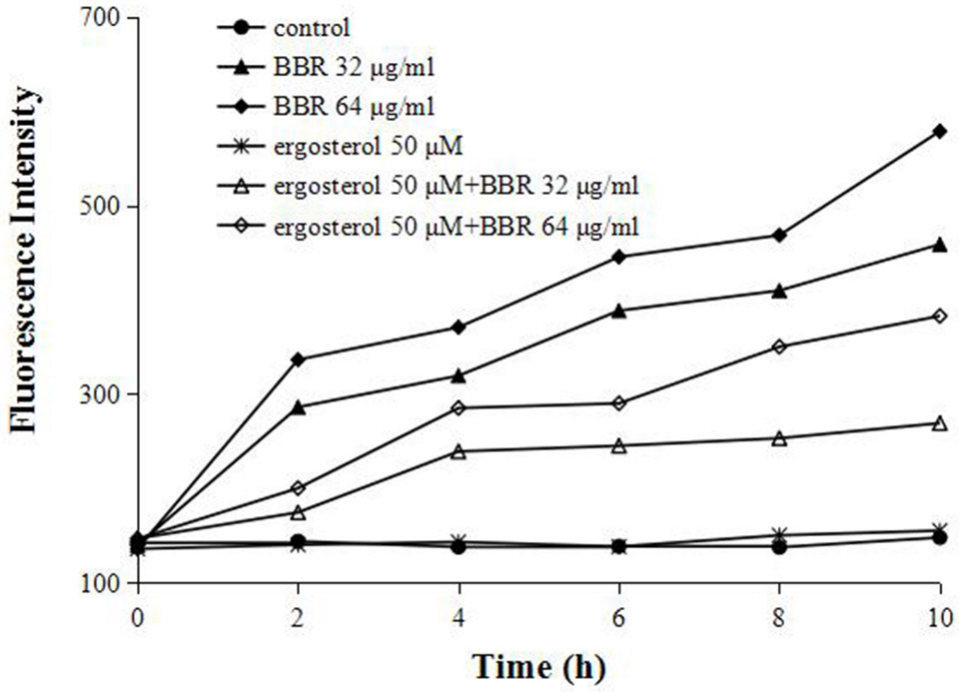
Ergosterol decreased the intracellular BBR concentration in *C. albicans* isolate 0304103. The cells were exposed to different concentrations of BBR and ergosterol. The fluorescence intensity was measured with an Infinite 200 Universal Microplate Reader at 405-nm excitation and 520-nm emission wavelengths.

To further test our hypothesis that the augmentation of ergosterol might contribute to the tolerance of FLC-resistant *C. albicans* to BBR, we evaluated the intracellular concentration of BBR with or without feeding exogenous ergosterol by fluorescence measurement. A strong fluorescence was detected in BBR-treated cells due to the higher treatment doses of BBR (32 and 64 μg/mL) (Figure 4). As expected, the feeding of 50 μM ergosterol markedly decreased the intracellular BBR fluorescence (Figure 4), indicating that the exogenous ergosterol could reduce the intracellular accumulation of BBR in FLC-resistant *C. albicans*.

### Feeding ergosterol upregulated drug efflux pump genes of BBR-treated *C. albicans* cells

**Figure 5:**
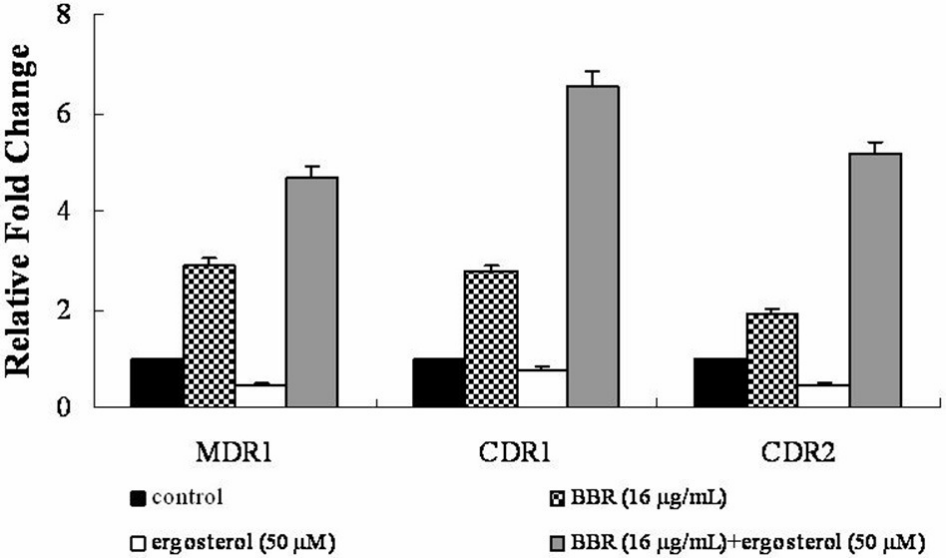
Changes in the expression of drug efflux pump genes *MDR1, CDR1*, and *CDR2* in *C. albicans* isolate 0304103 exposed to BBR and/or ergosterol. The cells were exposed to 16 μg/mL BBR and/or 50 μM ergosterol for 6 h. All genes were examined by real-time RT-PCR with gene-specific primers. Values represent the mean ± SD of three replicates.

The efflux of intracellular BBR was reported to be associated with the drug efflux pump in many species (Maeng et al., 2002; Li et al., 2013; Budeyri et al., 2016). Therefore, the mRNA levels of drug efflux pump genes *MDR1, CDR1*, and *CDR2* were analyzed by real-time RT-PCR in isolate 0304103 exposed to 16 μg/mL BBR and/or 50 μM ergosterol for 6 h. The same volume of solvents (DMSO, Tween 80, and ethanol) was added to the untreated control group. Figure 5 shows that BBR treatment upregulated the expression of genes *MDR1, CDR1*, and *CDR2* by 2.90-, 2.77-, and 1.92-fold, respectively. However, ergosterol treatment downregulated the expression of genes *MDR1, CDR1*, and *CDR2* by 56, 25, and 55%, respectively. Notably, the expression of these three genes significantly increased after BBR–ergosterol combination treatment (4.67-, 6.54-, and 5.17-fold increase for gene *MDR1, CDR1*, and *CDR2*, respectively). The results suggested that feeding ergosterol could further upregulate the drug efflux pump genes to increase the efflux of intracellular BBR, which might be one of the reasons for the decrease in intracellular BBR concentration in FLC-resistant *C. albicans* after exposure to exogenous ergosterol.

### Susceptibility of FLC-resistant *C. albicans* isolates to BBR was elevated by antifungals acting on ergosterol

Since the major antifungal mechanism of action of FLC was its inhibitory effect on cellular ergosterol synthesis, we postulated that FLC could sensitize the FLC-resistant isolates to BBR by decreasing ergosterol content. To validate this hypothesis, the antifungal efficacy of BBR combined with other antifungal agents in both isolates 0304103 and 01010 was investigated using checkerboard microdilution assay. As shown in Table 2, all the antifungal agents inhibiting ergosterol biosynthesis or binding to ergosterol, including sulconazole (SCZ), miconazole (MCZ), ketoconazole (KCZ), itraconazole (ITZ), amphotericin B (AMB), and nystatin (NST), demonstrated synergism with BBR against both isolates (FIC index ranged from 0.03 to 0.27). However, the antifungal agents that did not act on ergosterol, including N-myristoyl transferase inhibitor (N-MY) and 5-flucytosine (5-FC) (Weston et al., 1998; Vermes et al., 2000), were not synergistic with BBR (FIC index ranging from 1.02 to 2.02). The results indicated that the susceptibility of FLC-resistant *C. albicans* to BBR could be increased by antifungals acting on ergosterol, well supporting our hypothesis.

**Table 2:** Interaction of antifungal agents and BBR against *C. albicans* isolates 0304103 and 01010 detected using checkerboard microdilution assay.

**Parameter**	**SCZ**	**MCZ**	**KCZ**	**ITZ**	**AMB**	**NST**	**N-MY**	**5-FC**	**BBR**
**MIC_80_ (μg/mL) of isolate 0304103**									
Antifungal agent alone	128	32	128	2	1	4	8	0.03	>16
Combination with BBR^a^	0.06/1	0.06/1	0.06/8	0.03/2	0.03/1	0.25/1	8/0.5	0.05/0.5	
FIC index^b^	0.03	0.03	0.25	0.08	0.06	0.09	1.02	2.02	
**MIC_80_ (μg/mL) of isolate 01010**									
Antifungal agent alone	8	8	4	4	0.5	4	4	0.01	>16
Combination with BBR^a^	0.25/1	0.25/4	0.06/8	0.06/8	0.03/2	0.25/1	8/0.5	0.01/0.5	
FIC index^b^	0.06	0.16	0.27	0.27	0.13	0.09	2.02	1.02	

a *MIC_80_s for combinations are expressed as MIC_80_ of drug/MIC_80_ of BBR. High off-scale MIC_80_ was converted into the next highest concentration*.

b *Synergy and antagonism were defined by FIC indices of ≤ 0.5 and >4, respectively. An FIC index of >0.5 but ≤4 was considered indifferent (Odds, 2003)*.

### BBR promoted intracellular vacuole fusion

**Figure 6:**
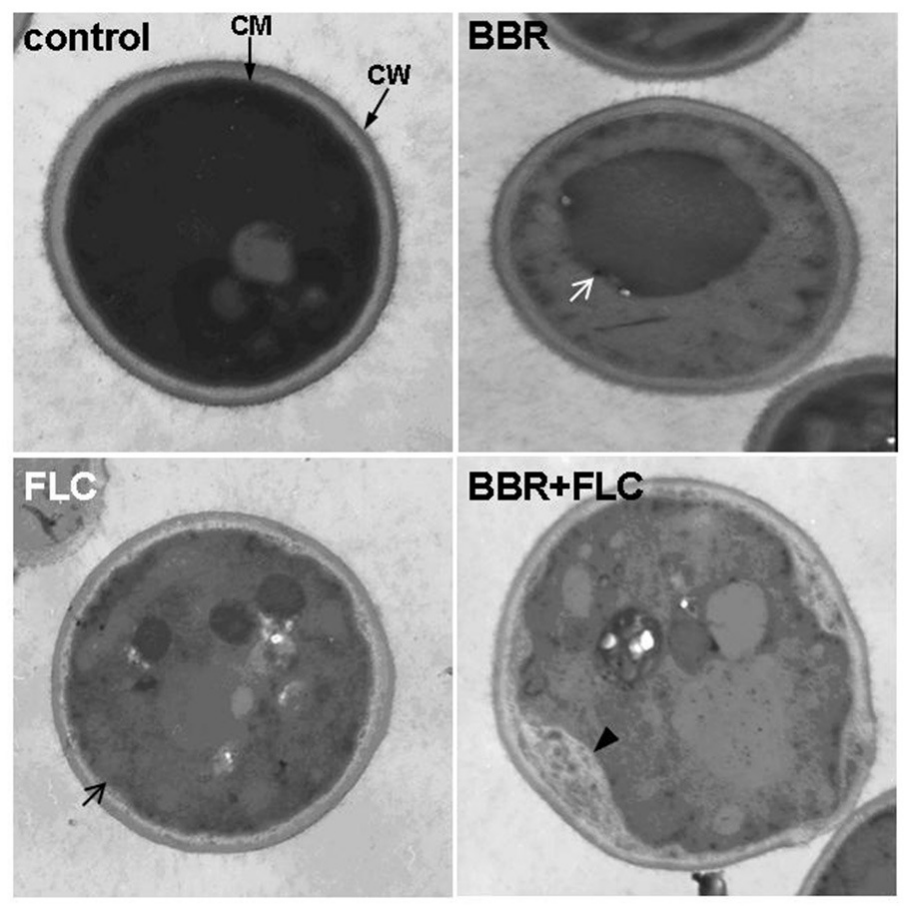
Cell ultrastructure of *C. albicans* isolate 0304103. Control, untreated cells; BBR, cells treated with 16 μg/mL BBR for 16 h; FLC, cells treated with 10 μg/mL FLC for 16 h; BBR + FLC, cells treated with 16 μg/mL BBR and 10 μg/mL FLC for 16 h. White arrow, intracellular vacuole; black arrow, damaged cell membrane; black arrowhead, detachment of the cell membrane from the cell wall.

Since ergosterol was also present in many other organelle membranes (Lv et al., 2016), the ultrastructural changes in the cells treated with BBR and/or FLC in isolate 0304103 were observed under an electron microscope. Figure 6 shows that the cells in the untreated control group were well preserved, with an intact cell wall, a normal-shape plasma membrane, and a homogeneous cytoplasm. Cells treated with 16 μg/mL BBR also exhibited normal morphology of cell wall and plasma membrane. Although isolate 0304103 was highly resistant to FLC, 10 μg/mL of FLC could modestly damage the cell membrane (black arrow). BBR + FLC treatment severely damaged the cells, as indicated by a detachment of the cell membrane from the cell wall (black arrow) and extensive solubilization in the cytoplasm. Interestingly, oversized vacuoles appeared in the cells subjected to BBR monotreatment (white arrow), indicating that the vacuoles fused after treatment with BBR. It was reported that the membrane fusion was enhanced in ergosterol-enriched vacuoles (Kato and Wickner, 2001; Jones et al., 2010; Tedrick et al., 2012). Therefore, the results demonstrated that BBR treatment could elevate the cellular ergosterol content in FLC-resistant *C. albicans*.

## Discussion

A large number of recent studies focused on the antifungal activities and the underlying mechanisms of action of BBR alone or in combination with other antifungal agents against *Candida* spp. (Dhamgaye et al., 2014; Da et al., 2016; Jing et al., 2016; Shi et al., 2017; Zorić et al., 2017). This study aimed to elucidate the mechanism underlying BBR tolerance in FLC-resistant *C. albicans* isolates and the effect of FLC on BBR tolerance. The results showed that BBR monotreatment induced increased expression of ergosterol synthesis genes and higher cellular ergosterol level, and also promoted fusion of intracellular vacuoles in clinical FLC-resistant isolates. Meanwhile, feeding exogenous ergosterol decreased intracellular BBR accumulation and increased the expression of genes related to drug efflux pump, further enhancing BBR tolerance. Additionally, only the antifungals reducing or binding to ergosterol could sensitize FLC-resistant *C. albicans* to BBR. The aforementioned results indicated that FLC-resistant *C. albicans* could tolerate BBR by promoting ergosterol synthesis, and BBR tolerance could be abolished by the antifungals acting on ergosterol. This explained the synergistic effect of FLC and BBR against FLC-resistant *C. albicans*.

Ergosterol, an important component of membrane lipids modulates the fluidity, permeability, and integrity of the membranes in *C. albicans*. These properties determine the susceptibility of *C. albicans* cells to a variety of stresses, such as ionic, osmotic, and oxidative pressures, and antifungal drug treatment (Miao et al., 2002; Casalinuovo et al., 2004; Shrivastava and Chattopadhyay, 2007; Jin et al., 2008). Besides the plasma membrane, the endomembrane system, including peroxisomes, mitochondria, vacuoles, and endoplasmic reticulum, also contains ergosterol (Schneiter et al., 1999), indicating that the ergosterol probably is involved in multiple biological activities in *C. albicans* cells (Lv et al., 2016). Our previous study demonstrated that FLC could increase the intracellular BBR concentration by causing damage to the plasma membrane in FLC-resistant *C. albicans*. Considering the multiple biological functions of ergosterol, it is believed that the damage to the plasma membrane may be one of the consequences of the reduction of ergosterol content by FLC. Also, a reduction in ergosterol may also induce many other biological changes in cells to synergistically enhance the susceptibility of FLC-resistant *C. albicans* to BBR. In other words, the reduction of ergosterol content probably is the upstream mechanism underlying the disruption of BBR tolerance by FLC in FLC-resistant *C. albicans*.

Previous studies demonstrated relatively weak antibiotic properties of BBR due to drug efflux pumps (Maeng et al., 2002; Li et al., 2013; Budeyri et al., 2016). In *Escherichia coli*, for example, about 30% of the cells still remain robust after prolonged BBR exposure, which is due to the efflux of BBR through multidrug resistance pumps (Budeyri et al., 2016). This study found that feeding exogenous ergosterol to BBR-treated cells further decreased the intracellular BBR concentration and upregulated the drug efflux pump genes in *C. albicans*, indicating that the overexpression of drug efflux pump genes might be one of the ways that ergosterol participated in BBR tolerance in FLC-resistant *C. albicans*. Of note, the study also found that the drug efflux pump genes were downregulated in the cells exposed to ergosterol only. It is believed that this downregulation could be a negative feedback effect of feeding exogenous ergosterol in *C. albicans*. Moreover, ergosterol could increase the expression of drug efflux pump genes only in the presence of BBR.

The involvement of ergosterol not only is confined to the membranes but also forms the basis of maintaining the localization and activities of some enzymes within the membrane (Lv et al., 2016). In this study, transmission electron microscopy showed oversized vacuoles in the cells treated with BBR alone, indicating that BBR could promote vacuole fusion in FLC-resistant *C. albicans* cells. A number of studies showed that the homotypic vacuolar fusion was closely related to the vacuolar H^+^-ATPase (V-ATPase) (Peters et al., 2001; Bayer et al., 2003; Baars et al., 2007), whose function also critically required ergosterol (Zhang et al., 2010). The precise role of V-ATPase (a multisubunit, rotary proton pump) in homotypic vacuole fusion has not yet been determined (Coonrod et al., 2013). Some studies demonstrated that the H^+^-translocation/vacuole acidification function, rather than the physical presence, of V-ATPase promotes homotypic vacuole fusion in yeast (Ungermann et al., 1999; Coonrod et al., 2013). However, the conclusions of other studies are contradictory (Takeda et al., 2008; Strasser et al., 2011; Yann et al., 2016). In consideration of the current controversy, the specific correlation between BBR tolerance and V-ATPase in *C. albicans* requires further investigation.

In conclusion, the present study demonstrated that ergosterol was required for BBR tolerance in FLC-resistant *C. albicans*. BBR treatment increased the cellular ergosterol content and upregulated the ergosterol synthesis genes. Feeding exogenous ergosterol further reduced the susceptibility of FLC-resistant *C. albicans* to BBR by decreasing intracellular BBR concentration and increasing the expression of drug efflux pump genes. Moreover, FLC abolished BBR tolerance by inhibiting ergosterol synthesis. The findings provided new insights into the synergistic mechanisms of action of antifungal combinations.

## Author contributions

YX, HQ, YW, HZ, JS, and JX performed the experiments. NJ and YJ designed the study. YX analyzed the data and wrote the paper. All authors approved the manuscript for publication.

### Conflict of interest statement

The authors declare that the research was conducted in the absence of any commercial or financial relationships that could be construed as a potential conflict of interest.

## References

[B1] Avmeet K. Kasturi S. M. Ashok R. Rajendra P. (2002). *In vitro* low-level resistance to azoles in *Candida albicans* is associated with changes in membrane lipid fluidity and asymmetry. Antimicrob. Agents Chemother. 46, 1046–1052. 10.1128/AAC.46.4.1046-1052.2002 11897588 PMC127087

[B2] Baars T. L. Petri S. Peters C. Mayer A. (2007). Role of the V-ATPase in regulation of the vacuolar fission-fusion equilibrium. Mol. Biol. Cell 18, 3873–3882. 10.1091/mbc.E07-03-0205 17652457 PMC1995711

[B3] Ball A. R. Casadei G. Samosorn S. Bremner J. B. Ausubel F. M. Moy T. I. (2006). Conjugating berberine to a multidrug resistance pump inhibitor creates an effective antimicrobial. ACS Chem. Biol. 1, 594–600. 10.1021/cb600238x 17168555

[B4] Bayer M. J. Reese C. Bühler S. Peters C. Mayer A. (2003). Vacuole membrane fusion: V0 functions after trans-SNARE pairing and is coupled to the Ca^2+^-releasing channel. J. Cell Biol. 162, 211–222. 10.1083/jcb.200212004 12876274 PMC2172786

[B5] Bhadra K. Kumar G. S. (2011). Therapeutic potential of nucleic acid-binding isoquinoline alkaloids: binding aspects and implications for drug design. Med. Res. Rev. 31, 821–862. 10.1002/med.20202 20077560

[B6] Boberek J. M. Stach J. Good L. (2010). Genetic evidence for inhibition of bacterial division protein FtsZ by berberine. PLoS ONE 5:e13745. 10.1371/journal.pone.0013745 21060782 PMC2966414

[B7] Budeyri G. N. Avci F. G. Yoneten K. K. Alaybeyoglu B. Ozkirimli E. Sayar N. A. (2016). Response of *Escherichia coli* to prolonged berberine exposure. Microb. Drug Resist. 23, 531–544. 10.1089/mdr.2016.0063 27854150

[B8] Casalinuovo I. A. Di F. P. Garaci E. (2004). Fluconazole resistance in *Candida albicans*: a review of mechanisms. Eur. Rev. Med. Pharmacol. Sci. 8, 69–77. 15267120

[B9] Coonrod E. M. Graham L. A. Carpp L. N. Carr T. M. Stirrat L. Bowers K. (2013). Homotypic vacuole fusion in yeast requires organelle acidification and not the V-ATPase membrane domain. Dev. Cell 27, 462–468. 10.1016/j.devcel.2013.10.014 24286827 PMC4086684

[B10] Da S. A. R. Batista D. A. N. J. Da S. C. R. Sousa C. R. D. Anny C. S. R. Domingues F. D. (2016). Berberine antifungal activity in fluconazole-resistant pathogenic yeasts: action mechanism evaluated by flow cytometry and biofilm growth inhibition in *Candida* spp. Antimicrob. Agents Chemother. 60, 3551–3557. 10.1128/AAC.01846-15 27021328 PMC4879420

[B11] De l. F.-S. N. Salcedo-Hernández R. Alanís-Guzmán M. G. Bideshi D. K. Barboza-Corona J. E. (2007). A new rapid fluorogenic method for measuring bacteriocin activity. J. Microb. Methods 70, 196–199. 10.1016/j.mimet.2007.03.020 17481758

[B12] Dhamgaye S. Devaux F. Vandeputte P. Khandelwal N. K. Sanglard D. Mukhopadhyay G.. (2014). Molecular mechanisms of action of herbal antifungal alkaloid berberine, in *Candida albicans*. PLoS ONE 9:e104554. 10.1371/journal.pone.0104554 25105295 PMC4126717

[B13] Ettefagh K. A. Burns J. T. Junio H. A. Kaatz G. W. Cech N. B. (2011). Goldenseal (*Hydrastis canadensis* L.) extracts synergistically enhance the antibacterial activity of berberine via efflux pump inhibition. Planta Med. 77, 835–840. 10.1055/s-0030-1250606 21157683 PMC3100400

[B14] Gudlaugsson O. Gillespie S. Lee K. Berg J. V. Hu J. Messer S.. (2003). Attributable mortality of nosocomial candidemia, revisited. Clin. Infect. Dis. 37, 1172–1177. 10.1086/378745 14557960

[B15] Horn D. L. Neofytos D. Anaissie E. J. Fishman J. A. Steinbach W. J. Olyaei A. J.. (2009). Epidemiology and outcomes of candidemia in 2019 patients: data from the prospective antifungal therapy alliance registry. Clin. Infect. Dis. 48, 1695–1703. 10.1086/599039 19441981

[B16] Jin H. Mccaffery J. M. Grote E. (2008). Ergosterol promotes pheromone signaling and plasma membrane fusion in mating yeast. J. Cell Biol. 180, 813–826. 10.1083/jcb.200705076 18299351 PMC2265586

[B17] Jing S. Shi G. X. Wang T. M. Wu D. Q. Wang C. Z. (2016). Antiproliferation of berberine in combination with fluconazole from the perspectives of reactive oxygen species, ergosterol and drug efflux in a fluconazole-resistant *Candida tropicalis* isolate. Front. Microbiol. 7:1516 10.3389/fmicb.2016.01516 27721812 PMC5034683

[B18] Jones L. Tedrick K. Baier A. Logan M. R. Eitzen G. (2010). Cdc42p is activated during vacuole membrane fusion in a sterol-dependent subreaction of priming. J. Biol. Chem. 285, 4298–4306. 10.1074/jbc.M109.074609 20007700 PMC2836034

[B19] Kato M. Wickner W. (2001). Ergosterol is required for the Sec18/ATP-dependent priming step of homotypic vacuole fusion. EMBO J. 20, 4035–4040. 10.1093/emboj/20.15.4035 11483507 PMC149151

[B20] Li D. D. Xu Y. Zhang D. Z. Quan H. Mylonakis E. Hu D. D.. (2013). Fluconazole assists berberine to kill fluconazole-resistant *Candida albicans*. Antimicrob. Agents Chemother. 57, 6016–6027. 10.1128/AAC.00499-13 24060867 PMC3837902

[B21] Liang R. M. Cao Y. B. Fan K. H. Xu Y. Gao P. H. Zhou Y. J.. (2009). 2-Amino-nonyl-6-methoxyl-tetralin muriate inhibits sterol C-14 reductase in the ergosterol biosynthetic pathway. Acta Pharmacol. Sin. 30, 1709–1716. 10.1038/aps.2009.157 19915585 PMC4007502

[B22] Lv Q. Yan L. Jiang Y. (2016). The synthesis, regulation, and functions of sterols in *Candida albicans*: well-known but still lots to learn. Virulence 7, 649–659. 10.1080/21505594.2016.1188236 27221657 PMC4991322

[B23] Maeng H. J. Yoo H. J. Kim I. W. Song I. S. Chung S. J. Shim C. K. (2002). P-glycoprotein-mediated transport of berberine across Caco-2 cell monolayers. J. Pharm. Sci. 91, 2614–2621. 10.1002/jps.10268 12434406

[B24] Miao L. Nielsen M. Thewalt J. Ipsen J. H. Bloom M. Zuckermann M. J.. (2002). From lanosterol to cholesterol: structural evolution and differential effects on lipid bilayers. Biophys. J. 82, 1429–1444. 10.1016/S0006-3495(02)75497-0 11867458 PMC1301944

[B25] Nakamoto K. Sadamori S. Hamada T. (1990). Effects of crude drugs and berberine hydrochloride on the activities of fungi. J. Prosthetic Dentist. 64, 691–694. 10.1016/0022-3913(90)90298-Q 2079677

[B26] Odds F. C. (2003). Synergy, antagonism, and what the chequerboard puts between them. J. Antimicrob. Chemother. 52, 1. 10.1093/jac/dkg301 12805255

[B27] Park K. S. Kang K. C. Kim J. H. Adams D. J. Johng T. N. Paik Y. K. (1999). Differential inhibitory effects of protoberberines on sterol and chitin biosyntheses in *Candida albicans*. J. Antimicrob. Chemother. 43, 667–674. 10.1093/jac/43.5.667 10382888

[B28] Pereira G. C. Branco A. F. Matos J. A. Pereira S. L. Parke D. Perkins E. L.. (2007). Mitochondrially targeted effects of berberine [Natural Yellow 18, 5,6-dihydro-9,10-dimethoxybenzo(g)-1,3-benzodioxolo(5,6-a) quinolizinium] on K1735-M2 mouse melanoma cells: comparison with direct effects on isolated mitochondrial fractions. J. Pharmacol. Exp. Therap. 323, 636–649. 10.1124/jpet.107.128017 17704354

[B29] Peters C. Bayer M. J. Bühler S. Andersen J. S. Mann M. Mayer A. (2001). Trans-complex formation by proteolipid channels in the terminal phase of membrane fusion. Nature 409, 581–588. 10.1038/35054500 11214310

[B30] Pfaller M. A. Diekema D. J. (2007). Epidemiology of invasive candidiasis: a persistent public health problem. Clin. Microbiol. Rev. 20, 133–163. 10.1128/CMR.00029-06 17223626 PMC1797637

[B31] Quan H. Cao Y. Y. Xu Z. Zhao J. X. Gao P. H. Qin X. F.. (2006). Potent *in vitro* synergism of fluconazole and berberine chloride against clinical isolates of *Candida albicans* resistant to fluconazole. Antimicrob. Agents Chemother. 50, 1096–1099. 10.1128/AAC.50.3.1096-1099.2006 16495278 PMC1426442

[B32] Samosorn S. Tanwirat B. Muhamad N. Casadei G. Tomkiewicz D. Lewis K.. (2009). Antibacterial activity of berberine-NorA pump inhibitor hybrids with a methylene ether linking group. Bioorg. Med. Chem. 17, 3866–3872. 10.1016/j.bmc.2009.04.028 19419877 PMC2759347

[B33] Schmitt M. E. Brown T. A. Trumpower B. L. (1990). A rapid and simple method for preparation of RNA from *Saccharomyces cerevisiae*. Nucleic Acids Res. 18, 3091–3092. 10.1093/nar/18.10.3091 2190191 PMC330876

[B34] Schneiter R. Brügger B. Sandhoff R. Zellnig G. Leber A. Lampl M.. (1999). Electrospray ionization tandem mass spectrometry (ESI-MS/MS) analysis of the lipid molecular species composition of yeast subcellular membranes reveals acyl chain-based sorting/remodeling of distinct molecular species en route to the plasma membrane. J. Cell Biol. 146, 741–754. 10.1083/jcb.146.4.741 10459010 PMC2156145

[B35] Shi G. Shao J. Wang T. Wu D. Wang C. (2017). Mechanism of berberine-mediated fluconazole-susceptibility enhancement in clinical fluconazole-resistant *Candida tropicalis* isolates. Biomed. Pharmacother. 93, 709–712. 10.1016/j.biopha.2017.06.106 28700974

[B36] Shrivastava S. Chattopadhyay A. (2007). Influence of cholesterol and ergosterol on membrane dynamics using different fluorescent reporter probes. Biochem. Biophys. Res. Commun. 356, 705–710. 10.1016/j.bbrc.2007.03.032 17374525

[B37] Stermitz F. R. Lorenz P. Tawara J. N. Zenewicz L. A. Lewis K. (2000). Synergy in a medicinal plant: antimicrobial action of berberine potentiated by 5'-methoxyhydnocarpin, a multidrug pump inhibitor. Proc. Natl. Acad. Sci. U.S.A. 97, 1433–1437. 10.1073/pnas.030540597 10677479 PMC26451

[B38] Strasser B. Iwaszkiewicz J. Michielin O. Mayer A. (2011). The V-ATPase proteolipid cylinder promotes the lipid-mixing stage of SNARE-dependent fusion of yeast vacuoles. EMBO J. 30, 4126–4141. 10.1038/emboj.2011.335 21934648 PMC3199395

[B39] Takeda K. Cabrera M. Rohde J. Bausch D. Jensen O. N. Ungermann C. (2008). The vacuolar V1/V0-ATPase is involved in the release of the HOPS subunit Vps41 from vacuoles, vacuole fragmentation and fusion. FEBS Lett. 582, 1558–1563. 10.1016/j.febslet.2008.03.055 18405665

[B40] Tedrick K. Trischuk T. Lehner R. Eitzen G. (2012). Enhanced membrane fusion in sterol-enriched vacuoles bypasses the Vrp1p requirement. Mol. Biol. Cell 15, 4609–4621. 10.1091/mbc.E04-03-0194 15254266 PMC519153

[B41] Ungermann C. Wickner W. Xu Z. (1999). Vacuole acidification is required for trans-SNARE pairing, LMA1 release, and homotypic fusion. Proc. Natl. Acad. Sci. U.S.A. 96, 11194–11199. 10.1073/pnas.96.20.11194 10500153 PMC18010

[B42] Vermes A. Guchelaar H. J. Dankert J. (2000). Flucytosine: a review of its pharmacology, clinical indications, pharmacokinetics, toxicity and drug interactions. J. Antimicrob. Chemother. 46, 171–179. 10.1093/jac/46.2.171 10933638

[B43] Volleková A. Kost'Álová D. Kettmann V. Tóth J. (2003). Antifungal activity of Mahonia aquifolium extract and its major protoberberine alkaloids. Phytother. Res. Ptr 17, 834–837. 10.1002/ptr.1256 12916091

[B44] Weston S. A. Camble R. Colls J. Rosenbrock G. Taylor I. Egerton M.. (1998). Crystal structure of the anti-fungal target N-myristoyl transferase. Nat. Struct. Biol. 5, 213–221. 10.1038/nsb0398-213 9501915

[B45] White T. C. Holleman S. Dy F. Mirels L. F. Stevens D. A. (2002). Resistance mechanisms in clinical isolates of *Candida albicans*. Antimicrob. Agents Chemother. 46, 1704–1713. 10.1128/AAC.46.6.1704-1713.2002 12019079 PMC127245

[B46] Wilson L. S. Reyes C. M. Stolpman M. Speckman J. Allen K. Beney J. (2002). The direct cost and incidence of systemic fungal infections. Value Health 5, 26–34. 10.1046/j.1524-4733.2002.51108.x 11873380

[B47] Wisplinghoff H. Bischoff T. Tallent S. M. Seifert H. Wenzel R. P. Edmond M. B. (2004). Nosocomial bloodstream infections in US hospitals: analysis of 24,179 cases from a prospective nationwide surveillance study. Clin. Infect. Dis. 39, 1093–1093. 10.1086/421946 15306996

[B48] Xu Y. Wang Y. Yan L. Liang R. M. Dai B. D. Tang R. J.. (2009). Proteomic analysis reveals a synergistic mechanism of fluconazole and berberine against fluconazole-resistant *Candida albicans*: endogenous ROS augmentation. J. Proteome Res. 8, 5296–5304. 10.1021/pr9005074 19754040

[B49] Yadav R. C. Kumar G. S. Bhadra K. Giri P. Sinha R. Pal S.. (2005). Berberine, a strong polyriboadenylic acid binding plant alkaloid: spectroscopic, viscometric, and thermodynamic study. Bioorg. Med. Chem. 13, 165–174. 10.1016/j.bmc.2004.09.045 15582461

[B50] Yann D. Stefano V. Maria R. Ruta G. Andreas M. (2016). Organelle acidification negatively regulates vacuole membrane fusion *in vivo*. Sci. Rep. 6:29045 10.1038/srep29045 27363625 PMC4929563

[B51] Zavrel M. Hoot S. J. White T. C. (2013). Comparison of sterol import under aerobic and anaerobic conditions in three fungal species, *Candida albicans, Candida glabrata*, and *Saccharomyces cerevisiae*. Eukaryotic Cell 12, 725–738. 10.1128/EC.00345-12 23475705 PMC3647772

[B52] Zhang Y. Q. Gamarra S. Garciaeffron G. Park S. Perlin D. S. Rao R. (2010). Requirement for ergosterol in V-ATPase function underlies antifungal activity of azole drugs. PLoS Pathog. 6:e1000939. 10.1371/journal.ppat.1000939 20532216 PMC2880581

[B53] Zorić N. Kosalec I. Tomić S. Bobnjarić I. Jug M. Vlainić T.. (2017). Membrane of *Candida albicans* as a target of berberine. BMC Complement. Alternat. Med. 17:268. 10.1186/s12906-017-1773-5 28514949 PMC5436450

